# Complications during elective cataract surgery: did the COVID-19 lockdown affect outcomes of ophthalmic surgery?

**DOI:** 10.1186/s12886-023-03225-4

**Published:** 2023-12-06

**Authors:** Nicky H. Hulsmans, Rudy M. M. A. Nuijts, Richard H. C. Zegers

**Affiliations:** 1 Department of Ophthalmology, Diakonessenhuis Utrecht, Zeist, The Netherlands; 2 Present adress: Department of Gynaecology, St. Antonius Hospital, Nieuwegein, Utrecht, The Netherlands; 3 University Eye Clinic, Maastricht University Medical Center (MUMC+), Maastricht, The Netherlands; 4 Department of Ophthalmology, Amsterdam UMC, Location AMC, Meibergdreef 9, Amsterdam, AZ 1105 The Netherlands; 5 Oogkliniek Maastricht, Maastricht, The Netherlands

**Keywords:** Cataract, Complications, COVID-19, Lockdown, Surgery

## Abstract

**Purpose:**

One of the many consequences of the COVID-19 pandemic was a worldwide lockdown of ophthalmic surgery procedures for several months in 2020. The present study aims to answer the following question: does an intermission of cataract surgery for two months cause an increase in complication rates?

**Methods:**

In this retrospective clinical chart review, data was taken from Dutch cataract complication registration database that contains pre-, intra- and postoperative information of patients that underwent cataract surgery in the Netherlands. The amount as well as type of complications were extracted before and after the eight weeks surgical intermission period (SIP): six weeks before (SIP-6) and six weeks after this period (SIP+6) for the years 2016–2020.

**Results:**

A significant decrease in complication rates was found between SIP-6 and SIP+6 in 2020. When SIP+6 2020 is compared to SIP+6 2019, a significant reduction is found. Overall, a downward trend in complication rates was observed in the period 2016–2020.

**Conclusion:**

A two-months intermission of performing elective cataract surgery does not cause an increase in complications. In contrast, we observe a reduction of postoperative complications. This implicates that refraining from cataract surgery for two months might not compromise operative skills. The possible downward trend over the years can be partially explained by improved training, education and equipment, as well as an increased use of intracameral antibiotics during operation. Possible explanations for the reduction of complications after lockdown could be decreased time pressure as a consequence of a reduced number of operations at the restart of surgeries, and heightened awareness and cautiousness when resuming the operations.

## Introduction

In many countries, the COVID-19 pandemic has caused a lockdown that has affected society as well as health care in an unprecedented manner. During the first worldwide COVID-19 spike in the first half of 2020, most hospitals postponed elective surgeries in order to reserve scarce health care capacity and medical equipment for COVID-19 care [2]. Scheduled procedures including cataract operations came to a sudden halt that lasted for two months. After this enforced surgical intermission, operations were resumed.

On estimation over 180,000 cataract extractions are conducted each year in the Netherlands, making this procedure the most commonly performed ophthalmologic operation [3]. Concerns have been raised that a lengthy interruption in this relative routine operation could affect surgical skills negatively, since surgical breaks have been associated with a decline in technical skills [4–6]. Consequently, a loss of surgical skills then could increase complication rates. The present study aims to answer the following question: does an enforced intermission of cataract surgery for two months cause an increase in complication rates? We feel like this is an important question for physicians as well as patients, as more surgical lockdowns in the future cannot be ruled out.

## Methods

Dutch cataract complication registration (DCCR) is an online database that contains pre-, intra-, and postoperative information of patients that undergo cataract surgery in the Netherlands. Although entering data into DCCR is on a voluntary base, a mean of 93.3% (range 91.9–94.8) of Dutch ophthalmologists, including ophthalmology residents, have registered their patients between 2016 and 2020 (source: Netherlands Ophthalmological Society). Registration is anonymous for patients as well as for ophthalmologists and the system allows to compare one’s own individual surgical results to those of the national peer group.

Permission was attained from the board of the Netherlands Ophthalmological Society (NOG) and Netherlands Intraocular Implant Club (NIOIC) to extract and analyze data from the anonymized database of DCCR to conduct this research. For this type of clinical research, we did not need approvement of a Medical Ethical Committee. In our retrospective clinical chart review we collected data from the database of DCCR of intra- and postoperative complications during the first COVID-19 spike in the Netherlands. Data was extracted before and after the eight weeks surgical intermission period (SIP): six weeks before (SIP-6) (week 6–11; 2020) and six weeks (SIP+6) (week 20–25; 2020). Both the number as well as the type of complications were collected. Data collected from the similar periods in 2016–2019 was used to evaluate the normal variance of the overall complication rates.

All patient and surgical baseline characteristics are displayed with the mean or absolute percentage. No range is given due to minimal differences between the weeks within the given periods. The total amount of complications in SIP-6 and SIP+6 were calculated by summing up the total amount of complications of every week in these periods. To calculate complication rates, the total number of complications of SIP-6 and SIP+6 were divided by the amount of conducted surgeries in SIP-6 and SIP+6, respectively. The total complication rates of SIP-6 were compared with the complication rates of SIP+6 with a two-by-two contingency table and the Chi-squared test statistical analysis. Also, the total cataract surgery complication rate of SIP-6 in 2020 was compared to total complication rate in the same period in 2019, as a control to analyze if the complication rate, before the intermission, was within normal yearly variance.

Chi square tests were performed to statistically analyze data. Statistical differences were considered significant at a level of *p* < 0.05. All statistical tests were performed two sided and were performed with SPSS 27 (IBM Corp. in Armonk, NY).

## Results

Demographic characteristics of the patient population receiving elective cataract surgery in SIP-6 and SIP+6 2020 are presented in Table 1. Data from the same periods in 2019 are also presented. In the period SIP-6 2020 a total of 15,141 operations was performed and in the period SIP+6 15,544 operations were performed. In comparable periods in 2019 more surgeries were performed; 21,270 and 19,087 respectively. The type and amount of complications in the period SIP-6 and SIP+6 in 2019 and 2020 are presented in Table 2.

**Table 1:** Patient and operation characteristics of the cataract surgeries performed 6 weeks prior (SIP-6: week 6–11) to the surgery reduction period and 6 weeks after surgery reduction period (SIP+6: week 20–25) in 2020 and of week 6–11 and week 20–25 in 2019

	Cataract operations 2020		Cataract operations 2019	
Characteristic	SIP-6 Operations week 6–11 (*N* = 15,141)	SIP+6 Operations week 20–25 (*N* = 15,544)	Operations week 6–11 (*N* = 21,270)	Operations week 20–25 (*N* = 19,087)
Age (y)	73.2	72.8	72.9	72.85
Gender N (%)				
Male	6359 (42)	6684 (43)	9146 (43)	8207 (43)
Female	8782 (58)	8860 (57)	12,124 (57)	10,880 (57)
Risk factors for complications^a^ N (%)	1469 (9.7)	1648 (10.6)	2893 (13.6)	2310 (12.1)
Factors restricting patient’s vision^b^ N (%)	3392 (22.4)	3435 (22.1)	5169 (24.3)	4676 (24.5)
Postoperative BCVA^c^ 0.50 N(%)	14,596 (96.4)	15,047 (96.8)	20,483 (96.3)	18,381 (96.3)
Operations with improved vision, ≥ 1 line^c^ N (%)	14,277 (94.3)	14,720 (94.7)	20,079 (94.4)	18,075 (94.7)
Operations with absolute refractive error < 1(D) from target refraction N (%)	14,308 (94.5)	14,736 (94.8)	20,058 (94.3)	18,037 (94.5)
Average absolute error from target refraction	0.38	0.37	0.39	0.39

BCVA: best-corrected visual acuity

^a^Previous corneal refractive surgery, previous vitrectomy, mature cataract, pseudoexfoliation, corneal opacity, small pupil, remainder

^b^Glaucoma, age-related macular degeneration, diabetic retinopathy, amblyopia, uveitis, remainder

^c^Snellen chart

**Table 2:** Number and percentage of intraoperative and postoperative cataract surgery complications in 2019 and 2020 in period SIP-6 and SIP+6

	Cataract operations 2020		Cataract operations 2019	
Type of complication	SIP-6 Operations week 6–11 (*N* = 15,141)	SIP+6 Operations week 20–25 (*N* = 15,544)	Operations week 6–11 (*N* = 21,270)	Operations week 20–25 (*N* = 19,087)
**Intraoperative**				
Posterior capsular rupture with vitreous loss, N (%)	27 (0.18)	25 (0.16)	50 (0.24)	32 (0.17)
Posterior capsular rupture without vitreous loss, N (%)	25 (0.17)	25 (0.16)	39 (0.18)	46 (0.24)
Iris prolapse, N (%)	18 (0.12)	23 (0.15)	29 (0.14)	25 (0.13)
Zonulolysis, N (%)	16 (0.11)	23 (0.15)	30 (0.14)	30 (0.16)
Capsulorhexis tear out, N (%)	24 (0.16)	27 (0.17)	22 (0.10)	30 (0.16)
Dropped nucleus, N (%)	12 (0.08)	6 (0.04)	8 (0.04)	10 (0.05)
Others, N (%)	30 (0,20)	18 (0,12)	28 (0,13)	30 (0,16)
*Total, N (%)*	*152 (100)*	*147 (100)*	*206 (100)*	*203 (100)*
**Postoperative**				
Persistent corneal edema, N (%)	16 (0.11)	12 (0.08)	23 (0.11)	29 (0.15)
Cystoid macular edema, N (%)	71 (0.47)	48 (0.31)	94 (0.44)	79 (0.41)
Endophthalmitis, N (%)	2 (0.01)	3 (0.02)	6 (0.03)	2 (0.01)
Others, N (%)	76 (0.50)	48 (0.31)	74 (0.35)	78 (0.41)
*Total, N (%)*	*165 (100)*	*111 (100)*	*197 (100)*	*188 (100)*

**Fig. 1:**
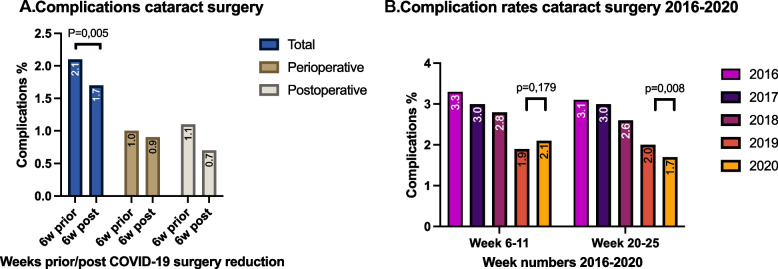
A Total, perioperative and postoperative cataract surgery complication rates prior to the surgery reduction period due to COVID-19 pandemic (SIP-6: week 6–11) and 6 weeks after surgery reduction period (SIP+6: week 20–25). B Cataract surgery complication rates 2016–2020 in weeks 6–11 and weeks 20–25

In SIP-6 2020 317 complications occurred; this complication rate of 2.1% consists of 152 (1.0%) intraoperative complications and 165 (1.1%) postoperative complications (Fig. 1A). In SIP+6 258 (1.7%) complications occurred, consisting of 147 (1.0%) intraoperative complications and 111 (0.7%) postoperative complications. The decrease in complication rate (-0.4%) between SIP-6 and SIP+6 is a significant difference (*p* = 0.005).

The complication rate of SIP-6 2020 is not statistically different when compared to the same period in 2019 (*p* = 0.179). However, when SIP+6 is compared to the same period in 2019, again a significant reduction is found (*p* = 0.008). Overall, a downward trend in complication rates can be observed in the period 2016–2020 (Fig. 1B).

## Discussion

It is postulated that a lengthy intermission of performing surgery, as happened in the first COVID-19 wave, could induce fading of surgical skills, hence resulting in increased complication rates. However, our results show that the enforced two-month surgical intermission at the peak of the COVID-19 pandemic in 2020 does not increase surgical complication rate when elective cataract operations were resumed.

Contrary to our expectations, a significant decrease in complication rate was observed compared to the period prior to the intermission, which seems to superimpose on the downward trend when benchmarking with previous years. Nevertheless, with the exception of the last two years, we have not taken in account the percentages of risk factors for complications, as well as for factors restricting patients' vision. Both percentages seem higher for the cases of 2019 compared to those in 2020. This can cause selection bias and hence can have had influence on the observed downward trend over the years. Since this latter aspect is beyond the scope of our research, we have not analyzed this any further. The possible decline in complication rates over the years might be partially explained by a better training of ophthalmologists and ophthalmology residents, technological perfection of equipment, and a reduction of infections that could be attributed to an increased use of intracameral antibiotics. The decrease in postoperative complications after the surgical lockdown seems to be mainly on account of the reduction in (corneal and macular) edema.

We hypothesize that the decrease of postoperative complications after surgical lockdown might be more COVID-related. The COVID-19 pandemic has caused changes in capacity management in hospitals, such as a reduction of the number of operations performed daily [7], thereby decreasing time pressure on ophthalmologists. Furthermore, the enforced intermission and consequent restart of surgeries might result in some sort of reset, including extra awareness and cautiousness, when resuming surgery. This is supported by a study of Tzamalis and colleagues who report an increased duration of cataract surgery, presumably due to ophthalmic surgeons being more careful upon the restart of surgery following the COVID-19 induced intermission [8]. Moreover, due to the COVID-19 pandemic hygiene practices were intensified, which could also have contributed to lower complication rates [9].

In accordance with our results, Tzamalis did not find a significant COVID-19-related change in intraoperative complication rates [8], although only the first 160 cases performed by eight consultants after the surgical lockdown were evaluated. Opposite to our findings, a study by Matarazzo and colleagues found an increased complication rate of posterior capsule ruptures (PCR) after the restart of elective cataract surgeries during the COVID-19 pandemic [10]. However, they only evaluated the complication rate of a single center while our research considers the performance of ophthalmologists working in virtually all hospitals in the Netherlands. More importantly, with an intermission period of 19 weeks, their study period of surgical abstinence was twice as long compared to our study. Theodoraki and colleagues in the United Kingdom looked at the incidence of PCR and postoperative cystoid macular edema (PCME) after two months of surgical lockdown in 2020 and evaluated an approximate 7-month period of operations after the lockdown [11]. They did not find a difference in PCR in these seven months, compared to an 11-month period prior to the pandemic. However, after a second surgical lockdown in January 2021, PCR rates were increased. This is in accordance with our results, but in contrast with the findings of Matarazzo [10]. In the study of Theodoraki, opposite to our findings, higher rates of PCME were noted after both surgical lockdowns (Fig. 1A) [11].

Note added in proof: recently, an American group independently obtained similar results [12]. Although there was a higher frequency of complex cataract surgeries performed post-shutdown, intraoperative complication rates before versus after the operation shutdown were not statistically significant. Nevertheless, this study contains a small sample size (306 eyes before and 174 eyes after shutdown were included).

An intermission of two months does not seem to compromise cataract surgical skills, which can be reassuring for both physicians and patients. A practical implication of our study could be that other causes for a leave of absence of two months, such as illness or a sabbatical, most likely will not negatively affect surgical performance. However, for a longer period the opposite may be true [10, 11].

There are some limitations of this study. Registration of complication data can be hampered by incomplete or incorrect data submission into the DCCR database. Cataract surgery combined with corneal, glaucoma or vitreoretinal procedures could not be excluded from the database. Furthermore, the DCCR does not include any information about the surgeon, hence it is not possible to differentiate between residents and ophthalmologists. It seems plausible that the consequences of a long-lasting interruption in surgical activities might have had a greater impact on the former group [5]. Nevertheless, the study of Das and colleagues (although their sample size being small) does not seem to confirm this assumption [12]. A survey enrolled in the United Kingdom found that especially residents reported to have reduced confidence and increased anxiety when restarting surgery after the forced COVID-19 surgical recess [13].

Future research might further investigate complication rates of individual cataract surgery complications to enhance awareness which complications might be more likely to occur after a surgical recess. In addition, it would be interesting to conduct similar research for other, non-ophthalmological elective surgical procedures which were postponed, to evaluate if comparable observations can be made in other surgical fields.

## Conclusion

The enforced intermission of performing elective cataract surgery, for a period of two months during the COVID-19 pandemic, did not result in an increase in complication rates. In contrast, we observe a reduction of postoperative complications. This implicates that refraining from cataract surgery for two months might not compromise operative skills. Explanations for the possible reduction of complications over the years, apart from patient and case selection, could be better training and education of ophthalmologists, introduction of devices with enhanced technological performance, and increased use of intracameral antibiotics. Explanations for the reduction of complications after a surgical lockdown could be decreased time pressure and heightened awareness and cautiousness.

## Data Availability

The data that support the findings of this study are available from the corresponding author upon reasonable request.
